# Evaluating the power of a recent method for comparing two circular distributions: an alternative to the Watson U^2^ test

**DOI:** 10.1038/s41598-023-36960-1

**Published:** 2023-06-20

**Authors:** Graeme D. Ruxton, E. Pascal Malkemper, Lukas Landler

**Affiliations:** 1 School of Biology, University of St Andrews, St Andrews, UK; 2 Research Group Neurobiology of Magnetoreception, Max Planck Institute for Neurobiology of Behavior – caesar, Ludwig-Erhard-Allee 2, 53175 Bonn, Germany; 3 Institute of Zoology, University of Natural Resources and Life Sciences (BOKU), 1180 Vienna, Austria

**Keywords:** Ecology, Physiology, Zoology, Ecology, Data processing, Software, Statistical methods

## Abstract

Some data are collected on circular (rather than linear) scales. Often researchers are interested in comparing two samples of such circular data to test the hypothesis that they came from the same underlying population. Recently, we compared 18 statistical approaches to testing such a hypothesis, and recommended two as particularly effective. A very recent publication introduced a novel statistical approach that was claimed to outperform the methods that we had indicated were highest performing. However, the evidence base for this claim was limited. Here we perform simulation studies to offer a more detailed comparison of the new “Angular Randomisation Test” (ART) with existing tests. We expand previous evaluations in two ways: exploring small and medium sized samples, and exploring a range of different shapes for the underlying distribution(s). We find that the ART controls type I error rates at the nominal level. The ART had greater power than established methods in detecting a difference in underlying distribution caused by a shift around the circle. Its performance advantage in this case was strongest when samples where small and unbalanced in size. When the difference between underlying unimodal distributions was in shape rather than central tendency, then the ART was at least as good (and sometimes considerably more powerful) than the established methods, except when distributions samples were small and uneven in size, and the smaller sample came from a more concentrated underlying distribution. In such cases its power could be markedly inferior to established alternatives. The ART was also inferior to alternatives in dealing with axially distributed data. We conclude that under widely-encountered circumstances the ART test can be recommended for its simplicity of implementation, but researchers should be aware of situations where it cannot be recommended.

## Introduction

Some variables (often related to orientations or timings) are recorded on circular scales. Such data need different statistical treatment from variables recorded on linear scales (see overviews in, for example, Mardia and Jupp^1^, Jammalamadaka and Sengupta^2^, and Ley and Verdebout^3^). A common question in circular statistics involves testing to see if two samples of circular data appear to come from different underlying distributions. For example, researchers interested in the effect of magnetic cues on the resting orientation of rodents might record the orientations of the long axis of some animals asleep under control conditions and some others under a manipulation of the prevailing magnetic field. Any substantial difference in these two samples might then be seen as evidence of magnetic sensitivity in rodents. Researchers have a wide choice of published methodologies for exploring this question statistically: recently we compared the performance of 18 such tests^4^. We concluded that two of these (Watson’s U^2^ test^5^ and a MANOVA approach^6^) could be recommended as controlling type I error rate near the nominal level and offering good statistical power over a broader range of situations than the other tests. Soon after the publication of our study, Ali and Abushilah^7^ published a novel angular randomisation test (ART) that they claimed was more powerful than the Watson’s U^2^ test. This would suggest that this new test might become the most attractive published so far and (combined with the simplicity of the test) this would argue for supporting its widespread uptake. Our aim here is to provide further exploration of the power and control of type I error rate of the ART. The investigations of Ali and Abushilah^7^ need to be expanded in two important ways. Firstly, they only explored the performance of their test for large sample sizes. The smallest single sample size considered was 100, which is an unrealistically high sample sizes for many fields of biological research. For example, in a survey of published studies on animal behaviour, Taborsky^8^ reported that the average sample sizes where 32 for field studies and 18 for studies based on captive animals. Secondly, they only considered a single shape of underlying distribution in their study, the von Mises distribution, which is a unimodal symmetrical bell-shaped distribution specified by two parameters (central location and dispersion, see e.g. Pewsey et al.^9^ for further discussion of its properties). However, a much broader range of distributions can occur, and the relative performance of tests can vary markedly with different underlying distributions (e.g., Landler et al.^4,10^). We will relax both these restrictions on the extent of investigations of the test here, as well as provide an easy-to-use R function for interested researchers to facilitate potential wider uptake of the ART.

## Materials and methods

### Defining the angular randomisation test (ART)

We assume that we record data in radian measure on a scale [0, 2*π*). We further assume that we have two samples of data of sizes m and n: {φ_1_, φ_2_, …. , φ_m_} and {ϕ_1_, ϕ_2_ , … , ϕ_n_}. Then the test statistic (*G*) is

$$G=\sum_{i=1}^{n}\sum_{j=1}^{m}D\left({\phi }_{i},{\psi }_{j}\right)$$

where $$D\left( {a,b} \right) = \pi - \left| {\pi - \left\lceil {a - b} \right\rceil } \right|$$.

*D* is the shortest angular (geodesic) distance between two points. Therefore, the test statistic is simply the sum of these distances from every point in one sample to every point in the other. The original formulation^7^ included a scalar multiplier, which we omit for brevity, since it would not influence our evaluation of the test.

To obtain the p-value associated with two samples we perform a permutation test. Firstly, we record the test statistic associated with the observed data (G*). We also attach a label to each data point associating it with either sample 1 or sample 2. We then produce a large number *N* of permutations of these *m* + *n* labels. For each permutation we can calculate a *G* value. If the number of permutations that produce a *G* value greater than G* is *Q*, then the *p* value is simply (*Q* + 1)/(*N* + 1). This is a standard way of carrying out a two-sample test by permutation—see Manly^11^, for example, for further discussion.

### Simulations

Our methods closely follow the approach we took in Landler et al.^4^. We compare the angular randomisation test with six other tests. Ali and Abushilah^7^ compared this test with tests using the same randomisation approach but the test statistics of Watson’s U^2^ test and Watson-Wheeler test. For these tests we obtain the test statistic from the implementation of those tests in the *circular* package in R. These implementations also provide p-values calculated using the analytic asymptotic version of the test statistic. In addition, we applied the recently proposed Rao spacing frequency test using the R code provided in Jammalamadaka et al.^12^. We call the six tests considered ART, WU2, pWU2, WW, pWW and Rsf, with the p suffix denoting the permutation version. In all cases we used 10,000 permutations.

We use the rcircmix function in the NPcirc package^13^ in R to produce either a unimodal von Mises, an axial von Mises (two modes on the opposite sides of the circle) or a wrapped skew-normal distribution (see Pewsey^14^ for a full description of the latter). We selected the wrapped skew-normal distribution because a previous investigation of tests of uniformity based on a single sample of circular data^10^ suggested that the relative performance of tests under this distribution was a good representation of their performance against plausible alternative skewed distributions. To specify a von Mises distribution two parameters need be specified: the mean (μ) and concentration parameter (*K*). *K* takes the value zero for a circular uniform distribution, with the distribution becoming increasingly concentrated for higher positive values of *K*. Three parameters are needed to specify a wrapped skew-Normal distribution: a location parameter (*ξ*), a dispersion parameter (*ρ*) and a shape parameter (*α*). The location parameter describes the central tendency of the distribution; whereas increasing (positive) values of the dispersion parameter indicate greater variance in values. Negative values of the shape parameter indicate a right skew; and positive values a left skew. The larger the magnitude of this parameter the stronger the skew (*α* = 0 indicates a symmetric distribution).

Having defined the parameters of the two underlying distributions, we report statistical power to detect a difference (or type I error rate for identical distributions) on the basis of 10,000 samples from the distributions. With 10,000 replicates, binomial theory suggests our estimated rates should be accurate to within 0.005.

## Results

**Figure 1:**
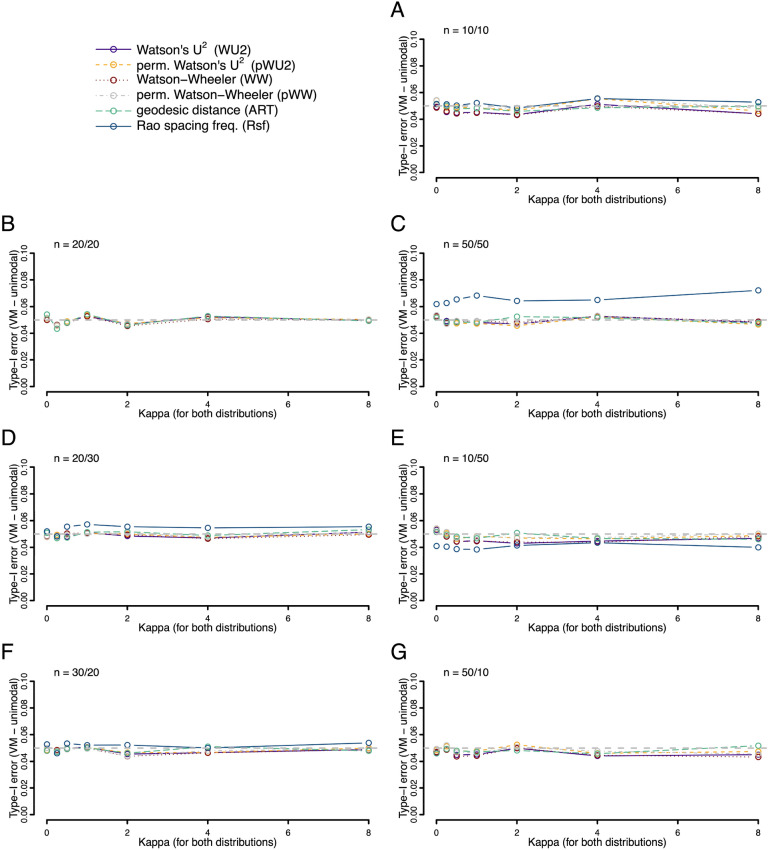
Type I error rates (fraction of occasions when the test incorrectly encourages the inference that the two samples are drawn from different distributions) for each of the six tests being compared. Both samples were drawn from the same von Mises (VM) distribution with a mean value of zero and a concentration given by the *K *value on the x-axis. Rates were calculated on the basis of 10,000 replicates. Different panels refer to different combinations of sample sizes.

**Figure 2:**
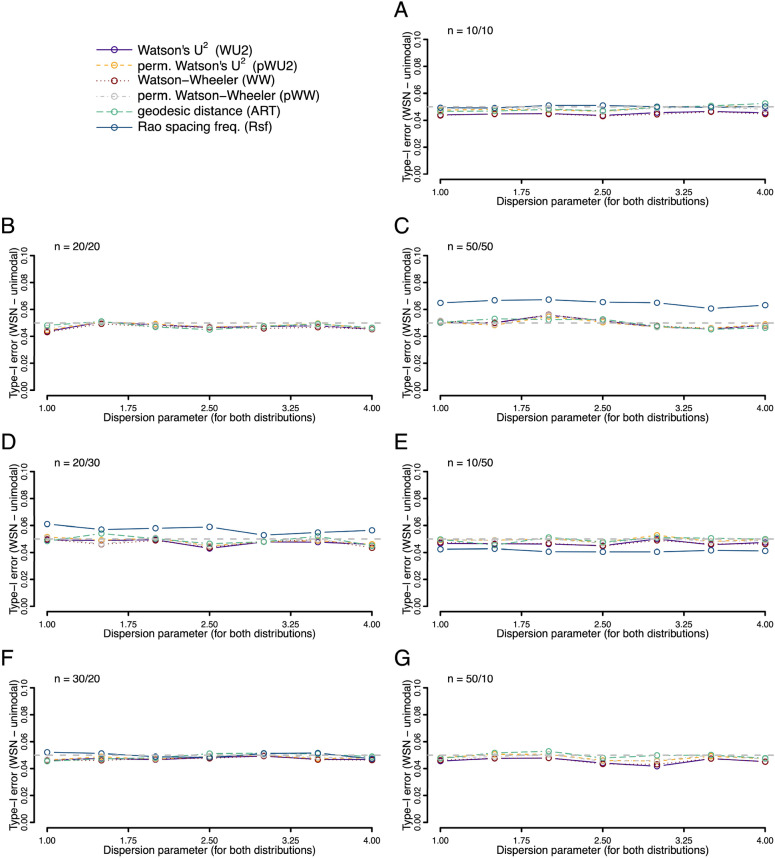
Type I error rates (fraction of occasions when the test incorrectly encourages the inference that the two samples are drawn from different distributions) for each of the six tests being compared. Both samples were drawn from the same wrapped skew normal (WSN) distribution with a mean value of zero, a shape parameter α of 30, and a dispersion given by the value on the x-axis. Rates were calculated on the basis of 10,000 replicates. Different panels refer to different combinations of sample sizes.

**Figure 3:**
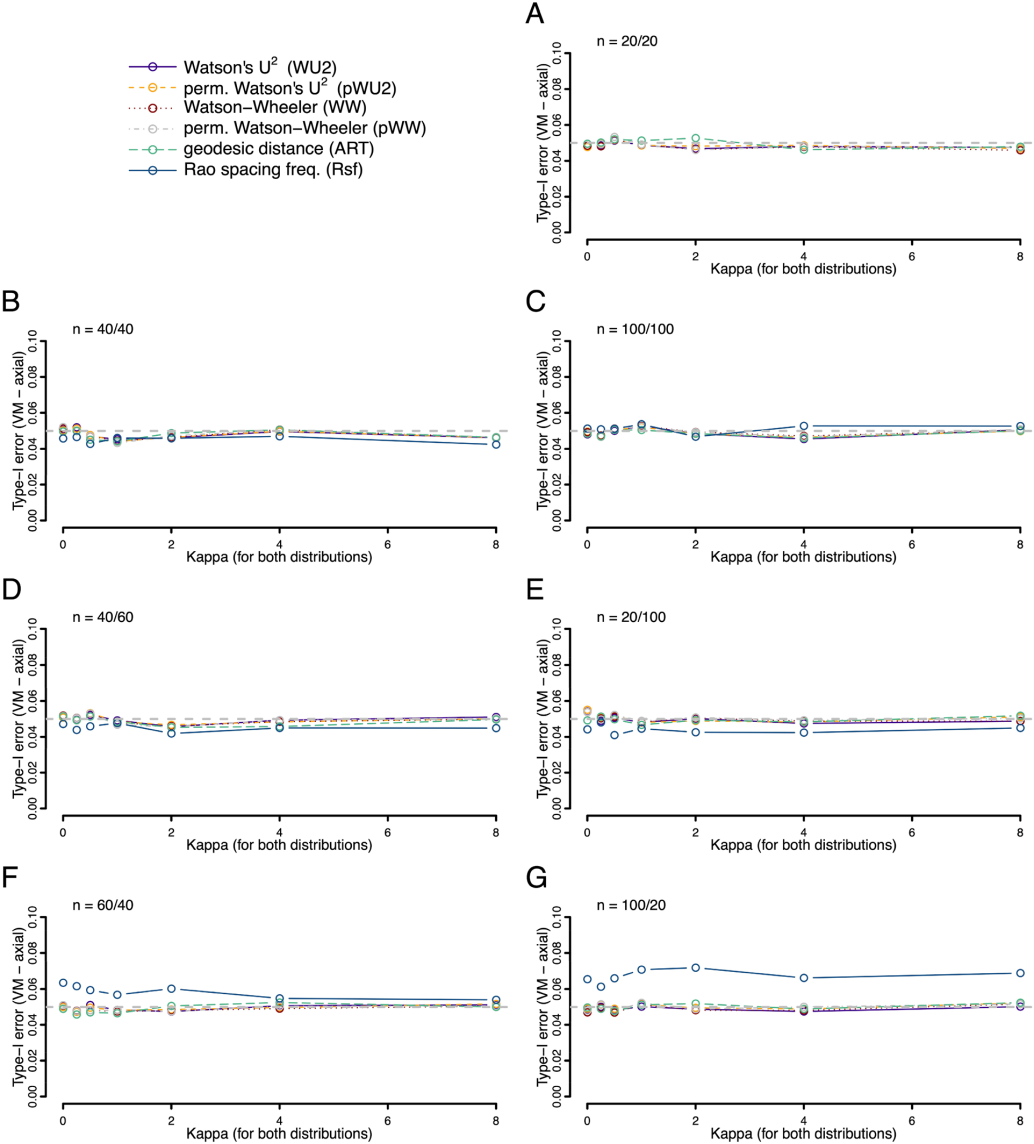
Type I error rates (fraction of occasions when the test incorrectly encourages the inference that the two samples are drawn from different distributions) for each of the six tests being compared. Both samples were drawn from the same axial von Mises (VM) distribution with the mean values of 0°/180° and a concentration given by the *K* value on the x-axis. Rates were calculated on the basis of 10,000 replicates. Different panels refer to different combinations of sample sizes.

The good control of type I error rate reported by Ali and Abushilah^7^ for ART using large samples from a von Mises distribution, held for small samples sizes too—even when the sample sizes were as small as ten in each, and even if sample sizes were strongly unbalanced (Fig. 1). This was true for a broad range of common concentration parameters (*K*) and for the majority of tests investigated (only the Rsf had slightly elevated type I errors in specific situations). We observed the same low type I error rates for identical skewed, as well as axial von Mises distributions (Figs. 2 and 3).

**Figure 4:**
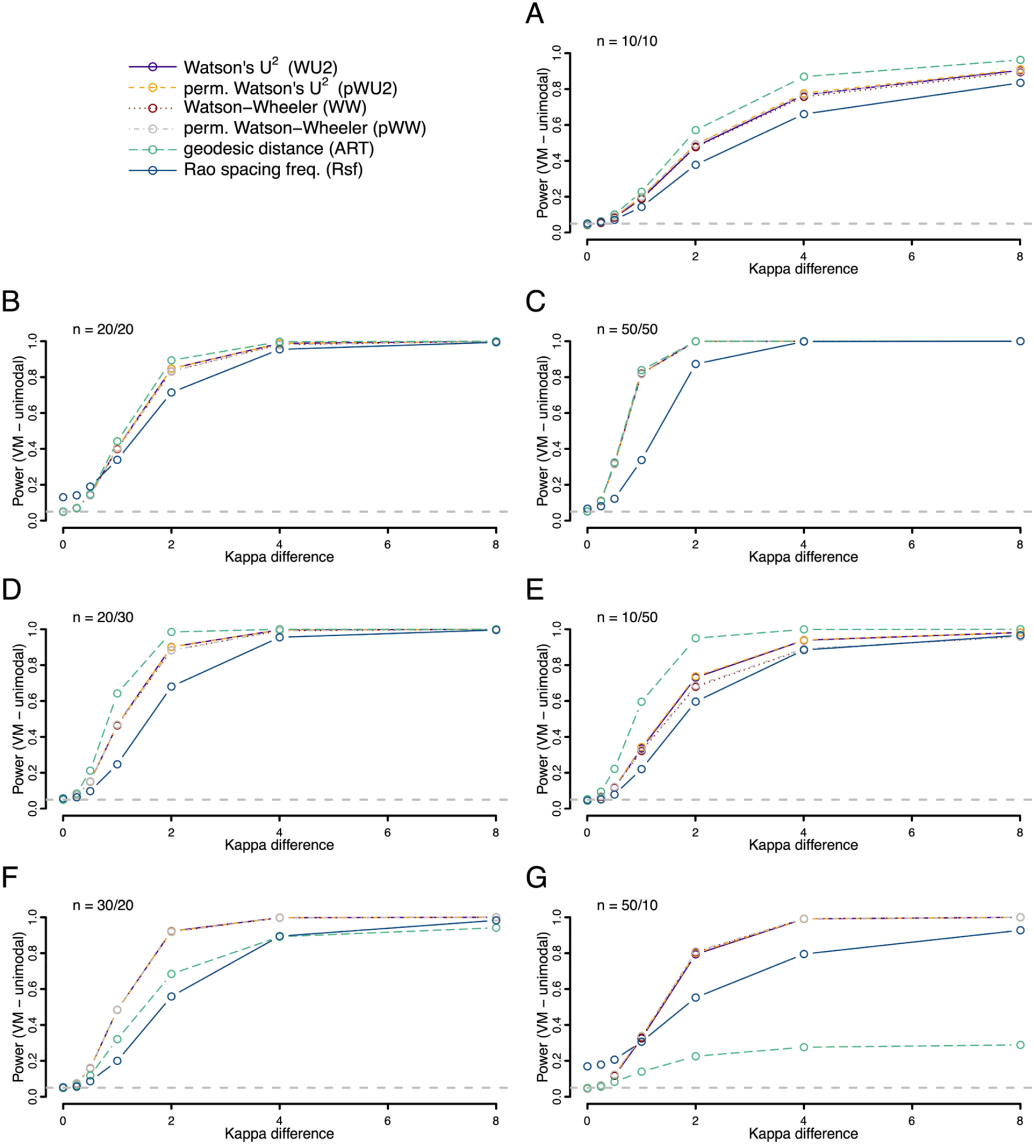
Statistical power (fraction of occasions when the test correctly encourages the inference that the two samples are drawn from different distributions) for each of the six tests being compared. Both samples were drawn from different unimodal von Mises (VM) distributions with a mean value of zero but different values of the concentration parameter (*K*). For the first sample kappa was always 0 (i.e., a uniform distribution), for the second the value ranged from 0 to 8 as given on the x-axis. Rates were calculated on the basis of 10,000 replicates. Different panels refer to different combinations of sample sizes.

**Figure 5:**
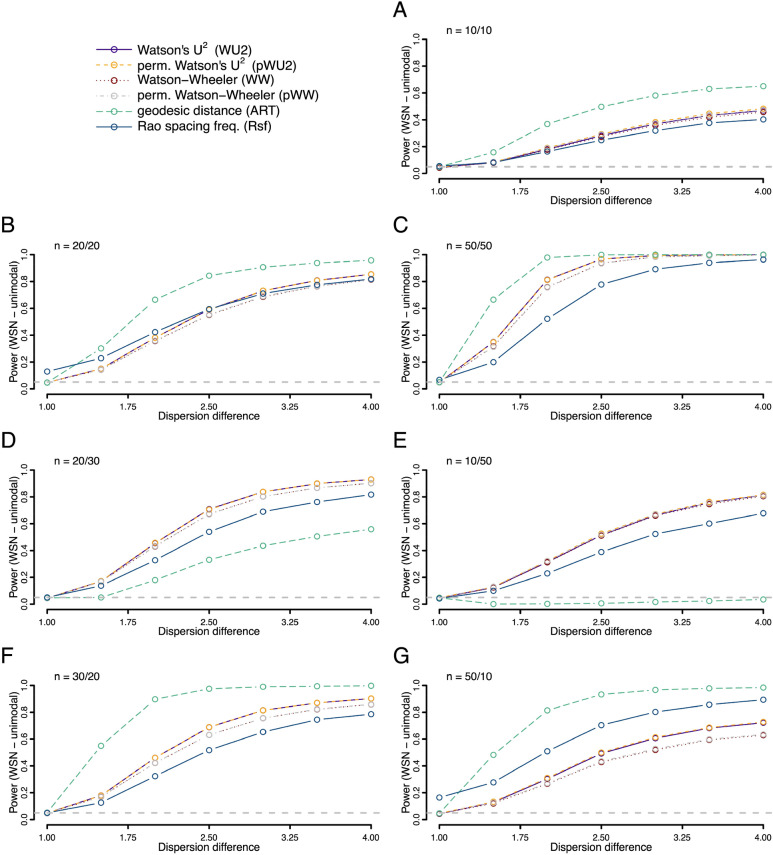
Statistical power (fraction of occasions when the test correctly encourages the inference that the two samples are drawn from different distributions) for each of the six tests being compared. Both samples were drawn from different unimodal wrapped skew normal (WSN) distributions with a mean value of zero and a shape parameter α of 30, but different values of the dispersion parameter. For the first sample one distribution was kept at the dispersion parameter ρ = 1 and for the second it ranged from 1 to 4 as given on the x-axis. Rates were calculated on the basis of 10,000 replicates. Different panels refer to different combinations of sample sizes.

**Figure 6:**
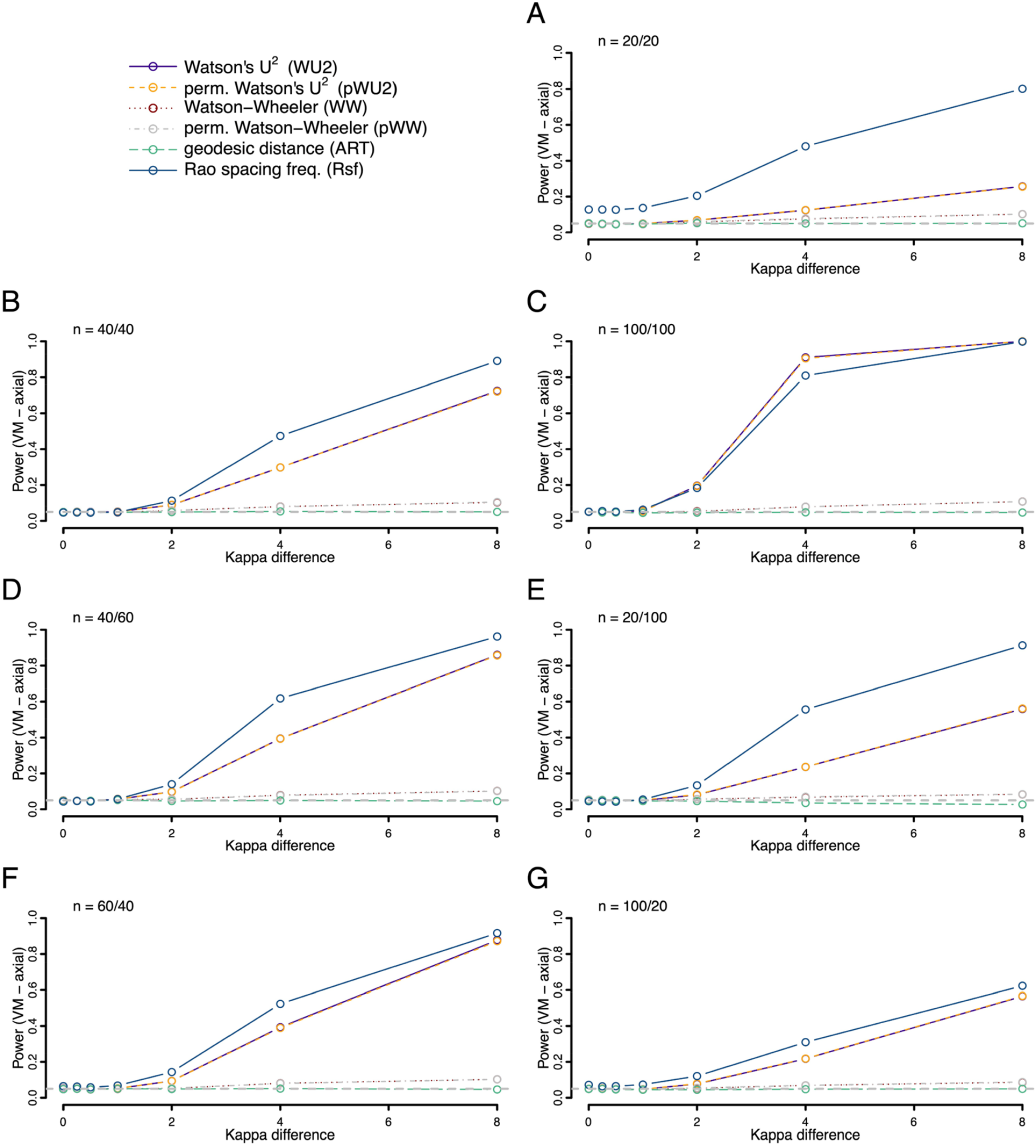
Statistical power (fraction of occasions when the test correctly encourages the inference that the two samples are drawn from different distributions) for each of the six tests being compared. Both samples were drawn from different axial von Mises (VM) distributions with mean values of 0°/180° but different values of the concentration parameter (*K*). For the first sample kappa was always 0 (i.e., a uniform distribution), for the second the value ranged from 0 to 8 as given on the x-axis. Rates were calculated on the basis of 10,000 replicates. Different panels refer to different combinations of sample sizes.

We further explored the power to detect an underlying difference in dispersion (spread) of the data points when the mean values of two underlying von-Mises distributions were identical (Fig. 4). Here, the ART offered more power than the other tests when sample sizes were small and balanced, i.e., when sample sizes were comparable. In unbalanced cases power was low when the higher dispersed sample had the higher sample size. The performance was similar for skewed data. Here the ART offered superior performance for balanced samples sizes, but substantially less power for unbalanced sample sizes, if the smaller sample had lower dispersion (Fig. 5). This drop in performance was most dramatic in the most uneven situation tested, with sample sizes of 10 (with low dispersion) and 50 (with high dispersion), where the ART offered close to zero power. The power for differences between samples drawn from axial von-Mises distributions (same mean values, different concentration), showed low power overall with superior power of the Rfs and no usable detection rate of the ART (Fig. 6).

**Figure 7:**
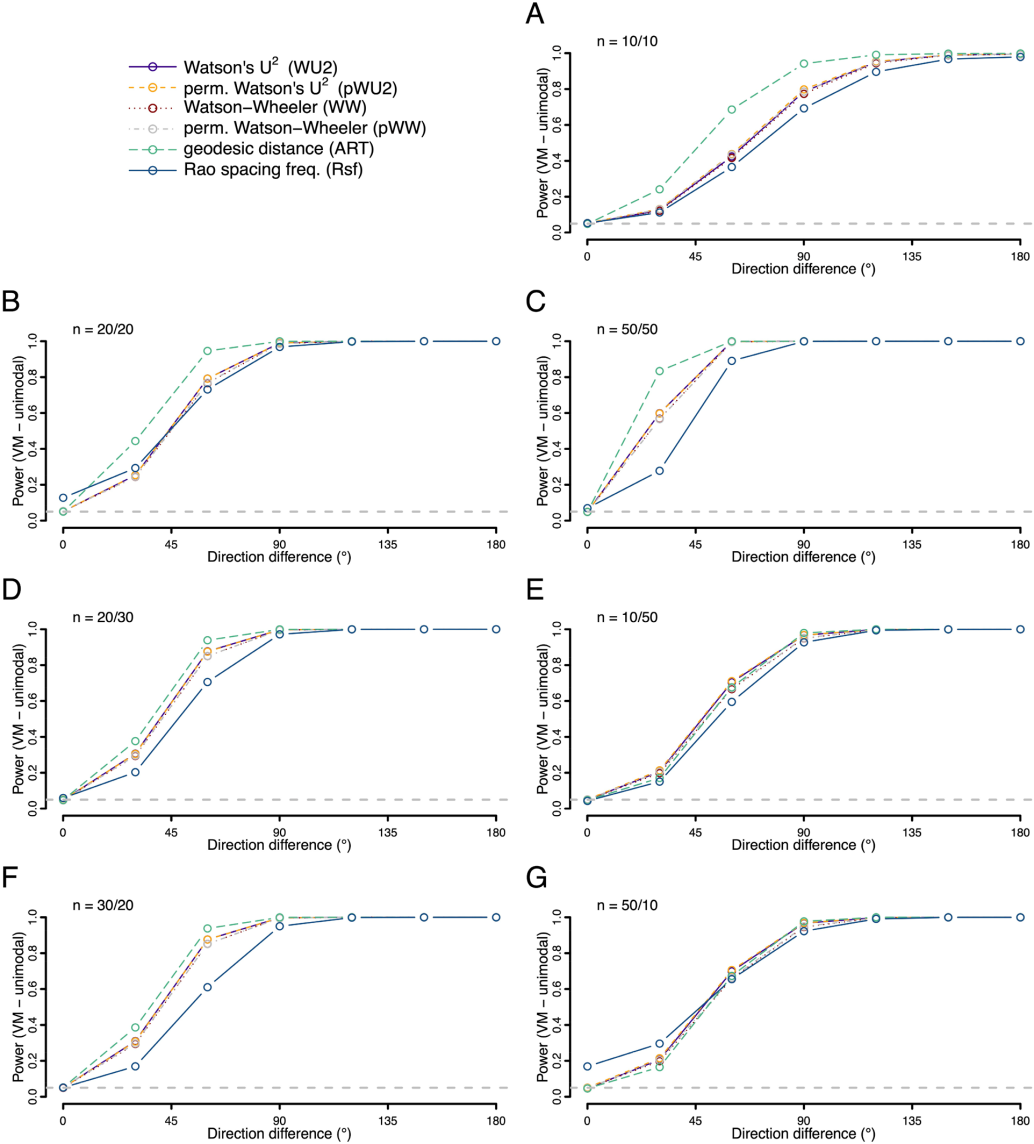
Statistical power (fraction of occasions when the test correctly encourages the inference that the two samples are drawn from different distributions) for each of the six tests being compared. Both samples were drawn from different unimodal von Mises (VM) distributions with the same concentration (*K* = 2) but different mean directions. For the first sample the mean direction was fixed at 0°, for the second the value ranged from 0° to 180° as given on the x-axis. Rates were calculated on the basis of 10,000 replicates. Different panels refer to different combinations of sample sizes.

**Figure 8:**
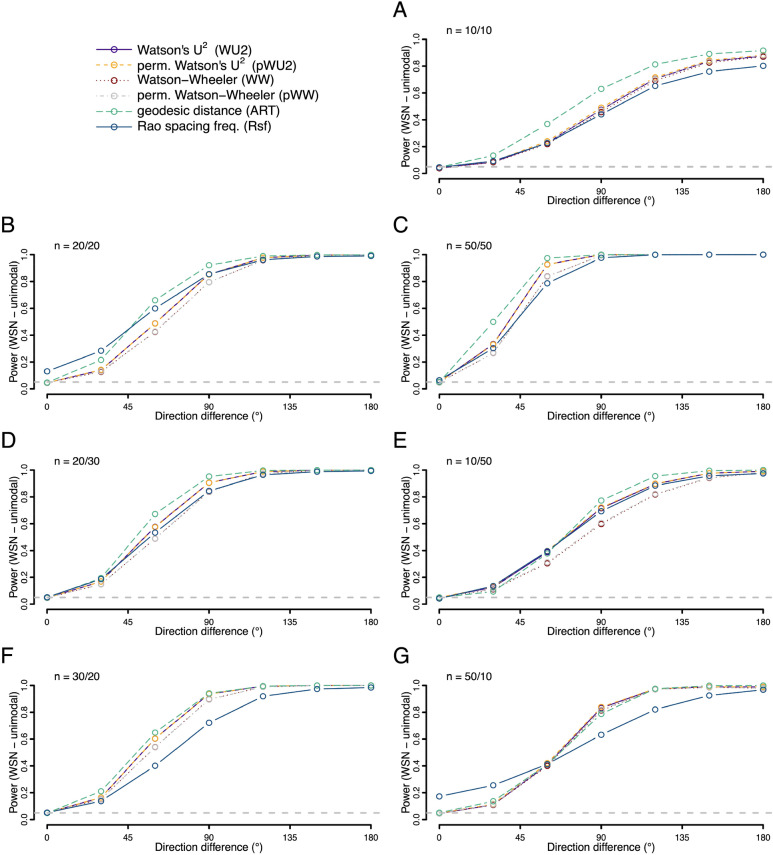
Statistical power (fraction of occasions when the test correctly encourages the inference that the two samples are drawn from different distributions) for each of the six tests being compared. Both samples were drawn from different unimodal wrapped skew normal (WSN) distributions with the same dispersion (ρ = 2) and shape (α = 30) but different mean directions. For the first sample the mean direction was fixed at 0°, for the second the value ranged from 0° to 180° as given on the x-axis. Rates were calculated on the basis of 10,000 replicates. Different panels refer to different combinations of sample sizes.

**Figure 9:**
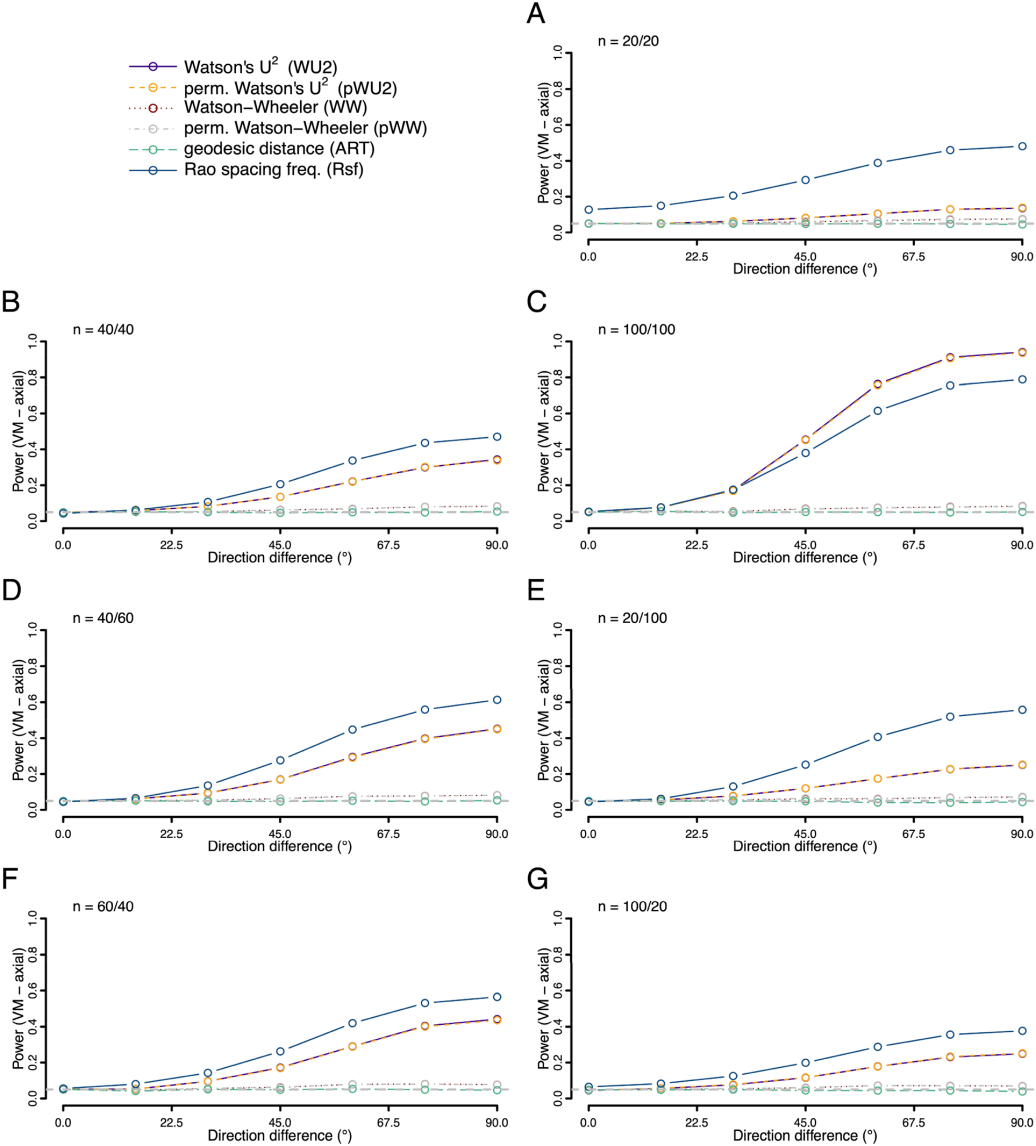
Statistical power (fraction of occasions when the test correctly encourages the inference that the two samples are drawn from different distributions) for each of the six tests being compared. Both samples were drawn from different axial von Mises (VM) distributions with the same concentration (*K* = 2) but different mean directions. For the first sample the mean directions were fixed at 0°/180°, for the second the values ranged from 0°/180° to 90°/270° as given on the x-axis (as the difference between the modes). Rates were calculated on the basis of 10,000 replicates. Different panels refer to different combinations of sample sizes.

We also explored the situation where both distributions had the same shape—but one was shifted around the circle relative to the other (Figs. 7, 8 and 9). Here we find the ART was overall the most powerful test for both unimodal symmetric and skewed distributions, with the most pronounced power advantage when sample sizes were small and balanced. However, for axial distributions the Rsf showed best power, with unusable power levels for ART (Fig. 9).

## Discussion

Here, we have evaluated the power of a recently proposed statistical test for the comparison of two circular samples, the ART^7^. For the most part we were able to strengthen the foundation for arguing for greater uptake of the ART (at least when underlying distributions are expected to be unimodal). Specifically, we show that it offers good control of type I error rate even if sample sizes are small, and/or the underlying distribution is quite different from a von Mises one. We also show that it offers good power in unimodal situations, regardless of whether the difference between underlying distributions is in central location or dispersion. Most importantly, the new test offers generally better power than the asymptotic and randomisation versions of Watson’s U^2^ test, the former of which was the joint winner in our comparison of 18 previously introduced tests^4^.

However, we have also uncovered two situations where the ART offers very poor or no power relative to alternatives. If samples are small and uneven in size, and the more dispersed sample is the larger sample, and the suspected difference in distributions is in dispersion (rather than shift), then the ART offers low power and cannot be recommended. If researchers find themselves in such a situation, then Watson’s U^2^ test can still be recommended. Also, for symmetrical bimodal (i.e., axial) distributions the ART offers almost no power and should not be used. Many commonly-used tests perform poorly in this situation^15^. This problem likely extends to other symmetric multimodal situations. The power of the ART for asymmetric multimodal situations has not been explored. Pending further exploration, we would not recommend the ART when underlying distributions are expected to be multimodal. In this case, the recently proposed Rao spacing frequency test described in Jammalamadaka et al.^12^ can be recommended. In other unimodal circumstances, our results and those of Ali and Abushilah^7^ argue that the ART is worthy of consideration for widespread uptake for the comparison of two circular distributions. There is no practical barrier to its implementation—we demonstrate above that its formulation is simple, and we offer an implementation in R here (Code can be downloaded at https://github.com/Malkemperlab/Geodesic-distance-test). The ART appears to offer more statistical power without any potential drawbacks in many standard situations, which should compel researchers to consider adding this novel test to their statistical repertoire.

Although we have developed the empirical support for the ART substantially over that offered by Ali and Abushilah^7^, there are certain unimodal situations we did not investigate. We do not know, for example, how the test behaves when faced with data rounded to a finite number of possible values (often called group data). However, similar tests seem relatively insensitive to even high levels of grouping^16^. Further, it may be possible to extend the methodology to compare more than two samples. Given the performance of the test in standard situations as reported here, such further explorations of its potential are warranted.

## Conclusions

We offer a considerably extended investigation of the properties of the recently introduced ART for comparing two samples of circular data. We conclude that under many circumstances the ART can be recommended for its simplicity of implementation combined with excellent control of type I error rate and power. Its power is generally superior to any of the previously introduced tests for this common research question. We caution, however, that we have uncovered situations where the newly introduced test has markedly poorer power than many previous tests—when underlying distributions are axially symmetric (or more generally symmetrically multimodal); or when underlying unimodal distributions vary in degree of concentration rather than location, samples are small and uneven in size, and the smaller sample comes from a more concentrated underlying distribution. If experimenters avoid these situations then uptake of this new test can be recommended.

## Data Availability

All code to rerun our analysis are available on https://github.com/Malkemperlab/Geodesic-distance-test.
